# Analysis of quality of life and risk factors in 122 patients with persistent hemodialysis

**DOI:** 10.12669/pjms.38.4.5308

**Published:** 2022

**Authors:** Jinhua Xie, Chuping Song

**Affiliations:** 1 Jinhua Xie, Department of Hemodialysis, Liyang Branch of Jiangsu Provincial People’s Hospital, 70 Jianshe West Road, Changzhou 213300, Jiangsu Province, P.R. China; 2 Chuping Song, Department of Hemodialysis, Liyang Branch of Jiangsu Provincial People’s Hospital, 70 Jianshe West Road, Changzhou 213300, Jiangsu Province, P.R. China

**Keywords:** Maintenance hemodialysis, Quality of life, Risk factors, SF-36

## Abstract

**Objectives:**

To summarize existing evidence on the quality of life and influencing factors in maintenance hemodialysis (MHD) patients.

**Methods:**

Total 122 MHD patients treated in the department of hemodialysis, Liyang branch of Jiangsu Provincial People’s Hospital from January 2018 to June 2020 were selected as the patient group. and 98 healthy persons in the same period were selected as the control group. The quality of life of the two groups was investigated by SF-36 score scale. At the same time, the clinical data of the patient group were investigated. Influencing factors of patients’ quality of life were analyzed by univariate test, and independent risk factors were analyzed by logistic multiple regression.

**Results:**

Univariate analysis showed that age, education, marital status, dialysis time, urea clearance index (Kt/V), income level, diabetic nephropathy, nutritional status, medical insurance type, depression and anxiety were single factors influencing the quality of life of MHD patients (P<0.05). Logistic multiple regression analysis showed that age ≥ 60 years, dialysis time ≥ one year, income less than 3000 yuan/month, primary disease is diabetic nephropathy., low medical insurance reimbursement, depression and anxiety were independent risk factors affecting the quality of life of MHD patients (P < 0.05).

**Conclusions:**

There was a relatively high proportion of patients with age ≥ 60 years, dialysis time ≥ one year, income less than 3000 yuan/month, primary disease is diabetic nephropathy, low medical insurance reimbursement, depression and anxiety. Timely nursing interventions such as reasonable nutritional support, comprehensive monitoring and psychological counseling for patients with the above situation may be helpful in improving quality of life of MHD patients.

## INTRODUCTION

With the change of health concept and medical model, the purpose of medical measures is not only to improve the outcome (survival time, survival rate, etc.), but also to improve quality of life of patients and their physical and mental health. Maintenance hemodialysis is a renal replacement therapy that uses semi permeable membrane to help uremic patients eliminate blood metabolic waste.^1^,^2^ Although this technology can significantly prolong the survival time of patients with chronic renal failure and improve the quality of life of patients to a certain extent, various chronic complications of dialysis, economic burden related to dialysis, limitations of patients’ normal physiological and social functions, emotional distress and other factors may have a serious impact on the quality of life of patients.^3^,^4^ Analyzing factors that may impact quality of life of MHD patients is crucial in order to establish timely and effective intervention measures. In this study, clinical data of 122 MHD patients were analyzed to identify independent risk factors affecting their quality of life.

## METHODS

One hundred twenty two MHD patients treated in the department of hemodialysis, Liyang branch of Jiangsu Provincial People’s Hospital from January 2018 to June 2020 were selected as the patient group, there were 65 males and 57 females; The age ranged from 22~80 years, with an average of (62.83±11.45) years, and the dialysis age ranged from 0.6~12 years, with an average of (2.9±1.4) years; The length of education is 0~16 years, with an average of (7.01±3.35) years. The primary disease was diabetic nephropathy (DN) in 56 cases and non DN in 66 cases.

### Inclusion criteria:

1) Age≥18 years; 2) Stable dialysis for more than six months; 3) Clear consciousness; 4) Signed informed consent and agreed to be included in this study.

### Exclusion criteria:

Patients with severe organ dysfunction, organic lesions and malignant tumors; Mental retardation. 98 healthy persons who underwent physical examination in the experience department in the same period were randomly selected into the control group, including 58 males and 40 females; The age ranged from 21~82 years, with an average of (64.10±12.30) years. The length of education was 0~19 years, with an average of (7.34±3.57) years. There was no significant difference in age, gender and education between the two groups (P>0.05).

The study was approved by the ethics committee of Liyang Branch of Jiangsu Provincial People’s Hospital (Approval number 2021098, Date: 2021-03-19).

The quality of life of MHD patients and control group was evaluated by using the international general health survey short form (SF-36 scale)^5^ developed by Boston Institute of health. The scale includes 8 dimensions and 36 items, including physiological function (PF), physiological function (RP), physical pain (BP), overall health (GH), vitality (VT), social function (SF), emotional function (RE) and mental health (MH), Each dimension is 0~100 points, and the final score is the average of the scores of the eight dimensions. The higher the score, the higher the quality of life of the patient.

Records of the clinical data for all MHD patients included gender, age, education, marital status, dialysis time, urea clearance index (Kt/V), income level, primary disease [diabetic nephropathy (DN) or non DN], nutrition status, medical insurance type, hemoglobin level, albumin level, depression and anxiety influencing factors.

Use SPSS 22.0 statistical software, the measurement data are expressed as mean±standard deviation (x̄±SD), and the mean of the two samples is compared by t-test. Independent risk factors affecting quality of life in MHD patients were analyzed by logistic multiple regression. P<0.05 was considered statistically significant.

## RESULTS

The scores of all dimensions of SF-36 in the patient group were significantly lower than those in the control group (P < 0.05) (Table-I). The results of univariate analysis showed that age, marital status, dialysis time, urea clearance index (Kt/V), income level, diabetic nephropathy, nutritional status, medical insurance type, depression and anxiety were single factors influencing the quality of life of MHD patients (P<0.05) (Table-II). Logistic multiple regression analysis showed that age ≥ 60 years, dialysis time ≥ one year, income less than 3000 yuan/month, primary disease is diabetic nephropathy, low amount of medical insurance reimbursement, depression and anxiety were independent risk factors affecting the quality of life scores of MHD patients (all P < 0.05) (Table-III).

**Table-I:** Comparison of quality of life between the two groups (score, x¯±SD).

Group	n	PF	RP	BP	GH	VT	SF	RE	MH
Patient group	122	48.14±17.49	37.54±18.70	49.95±17.73	48.19±15.75	46.67±16.22	48.8±16.32	52.8±16.79	59.73±17.48
Control group	98	81.03±20.45	70.84±15.76	81.44±16.28	65.7±16.65	70.47±15.93	85.35±15.32	76.63±13.03	75.33±11.45
t		12.850	14.327	13.704	7.985	10.928	17.080	11.846	7.955
P		<0.001	<0.001	<0.001	<0.001	<0.001	<0.001	<0.001	<0.001

**Table-II:** Single factor analysis of quality of life in MHD patients (x¯±SD).

Factor	Classification	n	SF-36 score	t	P
Gender	Male	65	48.40±15.82	0.452	0.652
	Female	57	49.73±16.79		
Age (years)	<60	54	62.00±10.19	11.451	<0.001
	≥60	68	38.72±12.26		
Years of Education (years)	≤9	94	45.29±14.97	5.105	<0.001
	>9	28	61.53±14.05		
Marital status	Married	51	52.90±17.37	2.274	0.025
	Single or Widowed	71	46.23±14.86		
Dialysis time (years)	<1	57	56.10±15.71	4.927	<0.001
	≥1	65	42.81±14.08		
Kt/V	<1.2	66	46.13±16.04	2.166	0.032
	≥1.2	56	52.42±15.92		
Income level (yuan/month)	<3000	60	41.31±13.13	5.818	<0.001
	≥3000	62	56.48±15.52		
Primary disease	Non DN	66	51.67±15.93	1.998	0.048
	DN	56	45.87±16.15		
Nutritional status	Good	64	52.65±15.98	2.660	0.009
	Bad	58	44.01±15.67		
Types of medical insurance	Medical insurance for urban residents	60	55.81±15.72	29.006	<0.001
	New rural cooperative medical insurance	62	42.45±13.93		
Hemoglobin (g/L)	<100	59	48.89±15.74	0.083	0.934
	≥100	63	49.14±16.80		
Albumin (g/L)	<3.5	41	49.78±15.83	0.365	0.716
	≥3.5	81	48.64±16.51		
Depression and anxiety	No	63	56.73±15.34	6.200	<0.001
	Yes	59	40.79±12.83		

**Table-III:** Logistic multiple regression analysis of the quality of life in patients with maintenance hemodialysis.

Factor	B	Standard error	t	P
Age	-12.596	3.043	4.131	<0.001
Dialysis time	-4.761	2.194	2.170	0.032
Income level	4.821	2.271	2.122	0.036
Primary disease	-5.355	1.974	2.713	0.008
Types of medical insurance	-5.164	2.331	2.216	0.029
Depression and anxiety	-4.849	2.289	2.118	0.036

## DISCUSSION

The modern medical model focuses on improving patient’s quality of life, as well as their physical and mental health.^6^ Numerous studies address the need to improve the quality of life of dialysis patients to reduce the burden on them, their families and society.^7^ Although maintenance hemodialysis can significantly prolong the survival time of patients, it cannot completely replace the kidney to remove toxins, improve metabolic disorders, or replace renal endocrine function. In the process of long-term treatment, factors such as age growth, physical dysfunction, increased economic burden and complications can seriously affect the quality of life and survival time of patients.^8^ Therefore, it is of great significance to analyze the related factors affecting the quality of life of MHD patients.^9^

In the MHD patients, serious degradation of bodily functions and self-care abilities, increase in complications, and the continuous development of corresponding symptoms, associated with the old age, seriously affect the quality of life. In a clinical study, Zhang L et al.^10^ showed that age is one of the main factors affecting quality of life of MHD patients. Our study demonstrated that old age (≥ 60 years) is an independent risk factor affecting the quality of life of MHD patients. This effect may be related to the decline of bodily functions, irreversible progress of accompanying symptoms and a decline in daily activity in elderly patients. Long-term dialysis treatment also has a serious impact on patients’ abilities to function independently, increasing their dependence on relatives, and reducing their social adaptability, potentially leading to anxiety and depression.^11^ The current study also shows that the time of analysis (≥ one year) is also one of the independent risk factors affecting the quality of life. Low income level and poor economic status of MHD patients themselves or their families make it difficult to maintain dialysis treatment for a long time, and to bear further treatment costs, affecting quality of life of patients themselves, and their family members.^12^ This study found that the incidence of SF-36 is low in patients with diabetic nephropathy. This is associated with diabetes mellitus as a basic disease complicated by renal dysfunction, which is associated with multiple organ dysfunction and cardiovascular, cerebrovascular and fundus lesions after reaching the dialysis stage, thus seriously affecting the survival quality of,^13^ which is consistent with the findings of Song KK et al.^14^ In recent years, with the deepening of the reform in China’s basic medical security system, influencing factors, such as medical expense payment composition, is attracting more and more attention.^15^ China’s social insurance is mainly divided into basic medical insurance for urban residents and new rural cooperative medical insurance, with a different degree of protection and reimbursement. China’s agricultural population base is large and the reimbursement amount of the new rural cooperative medical insurance is low. Therefore, the type of medical insurance is also closely related to the quality of life of patients.^16^ Depression and anxiety are common accompanying conditions in MHD patients. Studies show that depression and anxiety can affect immune function of patients, and can reduce their treatment compliance, thereby reducing overall quality of dialysis treatment.^17^ Depression and anxiety can also aggravate the decline of cognitive function, lead to heavier burden on family members, and negatively affect the family dynamics.^18^ Depression and anxiety, therefore, are important factors, contributing to the decline of quality of life in MHD patients.

### Limitations of the study:

However, due to the regional limitations, China’s vast territory and differences in economy and culture, this study is not representative of the overall effect of these factors on the quality of life of dialysis patients in China.

## CONCLUSION

Our study concludes that old age (≥ 60 years), dialysis time (≥ one year), income low than three thousand Yuan/month, primary disease is diabetic nephropathy, low reimbursement amount of medical insurance, depression and anxiety are the factors affecting quality of life of MHD patients.

### Authors’ contributions:

**JX:** Conceived, designed the study and is responsible for clinical integrity of the study.

**JX & CS:** Collected the data and performed the analysis.

**JX:** Was involved in the writing of the manuscript and also responsible for integrity of the study.

**CS:** Edited the manuscript, analysis and interpretation of data.

All authors have read and approved the final manuscript.
